# The European Prevalence of Resistance Associated Substitutions among Direct Acting Antiviral Failures

**DOI:** 10.3390/v14010016

**Published:** 2021-12-22

**Authors:** Stephanie Popping, Valeria Cento, Carole Seguin-Devaux, Charles A. B. Boucher, Adolfo de Salazar, Eva Heger, Orna Mor, Murat Sayan, Dominique Salmon-Ceron, Nina Weis, Henrik B. Krarup, Robert J. de Knegt, Oana Săndulescu, Vladimir Chulanov, David A. M. C. van de Vijver, Federico García, Francesca Ceccherini-Silberstein

**Affiliations:** 1 Department of Viroscience, Erasmus University Medical Center, 3015 CN Rotterdam, The Netherlands; c.boucher@erasmusmc.nl (C.A.B.B.); d.vandevijver@erasmusmc.nl (D.A.M.C.v.d.V.); 2 Department of Oncology and Hemato-Oncology, University of Milan, 20122 Milan, Italy; valeriacento@gmail.com; 3 Department of Infection and Immunity, Luxembourg Institute of Health, 4354 Esch sur Alzette, Luxembourg; Carole.Devaux@lih.lu; 4 The European Society for Translational Antiviral Research, 3584 CX Utrecht, The Netherlands; fegarcia@ugr.es (F.G.); ceccherini@med.uniroma2.it (F.C.-S.); 5 Department of Clinical Microbiology & Infectious Diseases, Instituto Bioanitario Ibs.Granada, Hospital Universitario San Cecilio, 18012 Granada, Spain; adolsalazar@gmail.com; 6 University Hospital Cologne, 50937 Cologne, Germany; eva.heger@uk-koeln.de; 7 Central Virology Laboratory, Ministry of Health, Sheba Medical Center, Ramat-Gan 52621, Israel; orna.mor@sheba.health.gov.il; 8 Sackler Faculty s of Medicine, Tel Aviv 69978, Israel; 9 Clinical Laboratory, Faculty of Medicine, Kocaeli University, Kocaeli 41380, Turkey; muratsayan@hotmail.com; 10 Research Center of Experimental Health Sciences, Near East University, Nicosia 99138, North Cyprus, Turkey; 11 Assistance Publique Hopitaux de Paris, University of Paris, 75004 Paris, France; dominique.salmon@cch.aphp.fr; 12 ESCMID Study Group for Viral Hepatitis (ESGVH), European Society of Clinical Microbiology and Infectious Diseases, 4100 Basel, Switzerland; 13 Department of Infectious Diseases, Copenhagen University Hospital Hvidovre, 2650 Hvidovre, Denmark; nina.weis@regionh.dk; 14 Department of Clinical Medicine, Faculty of Health and Medical Sciences, University of Copenhagen, 1165 Copenhagen, Denmark; 15 Department of Molecular Diagnostics, Aalborg University Hospital, 9100 Aalborg, Denmark; h.krarup@rn.dk; 16 Department of Clinical Medicine, Aalborg University, 9100 Aalborg, Denmark; 17 Department of Gastroenterology and Hepatology, Erasmus MC, University Medical Center, 3015 GD Rotterdam, The Netherlands; r.deknegt@erasmusmc.nl; 18 National Institute for Infectious Diseases “Prof. Dr. Matei Bals”, 021105 Bucharest, Romania; oana.sandulescu@umfcd.ro; 19 Department of Infectious Diseases I, Carol Davila University of Medicine and Pharmacy, 021105 Bucharest, Romania; 20 National Medical Research Center of Phthisiopulmonology & Infectious Diseases, 620039 Moscow, Russia; vladimir.chulanov@rcvh.ru; 21 Department of Infectious Diseases, Sechenov University, 119991 Moscow, Russia; 22 Scientific Center for Genetics and Life Sciences, Division of Biotechnology, Sirius University of Science and Technology, 354340 Sochi, Russia; 23 Department of Experimental Medicine and Surgery, University of Rome Tor Vergata, 00133 Rome, Italy

**Keywords:** hepatitis C, direct-acting antivirals, resistance, resistance associated substitutions, elimination

## Abstract

**Background:** Approximately 71 million people are still in need of direct-acting antiviral agents (DAAs). To achieve the World Health Organization Hepatitis C elimination goals, insight into the prevalence and influence of resistance associated substitutions (RAS) is of importance. Collaboration is key since DAA failure is rare and real-life data are scattered. We have established a European collaboration, HepCare, to perform in-depth analysis regarding RAS prevalence, patterns, and multiclass occurrence. **Methods:** Data were extracted from the HepCare cohort of patients who previously failed DAA therapy. Geno—and subtypes were provided by submitters and mostly based on in-house assays. They were reassessed using the Comet HCV subtyping tool. We considered RAS to be relevant if they were associated with DAA failure in vivo previously reported in literature. **Results:** We analyzed 938 patients who failed DAA therapy from ten different European countries. There were 239 genotypes (GT) 1a, 380 GT1b, 19 GT2c, 205 GT3a, 14 GT4a, and 68 GT4d infections. Several unusual subtypes (n = 15) (GT1b/g/l, GT3b, GT4k/n/r/t) were present. RAS appeared in over 80% of failures and over a quarter had three or more RAS. Multiclass RAS varied over target region and genotype between 0–48%. RAS patterns such as the Q30R + L31M and Q30R + Y93H in GT1a, the L31V + Y93H and L31V + Y93H for GT1b, and A30K + L31M and A30K/V + Y93H for GT3a all occurred with a prevalence below 5%. **Conclusion:** RAS occur frequently after DAA failures and follow a specific genotype and drug related pattern. Interpretation of the influence of RAS on retreatment is challenging due to various patterns, patients’ characteristics, and previous treatment history. Moving towards HCV elimination, an ongoing resistance surveillance is essential to track the presence of RAS, RAS patterns and gather data for a re-treatment algorithm.

## 1. Introduction

Approximately 71 million people worldwide are infected with the hepatitis C virus (HCV), from which only a small proportion received curative direct-acting antiviral agents (DAAs) [1]. In addition, the HCV epidemic is still ongoing in several population groups, such as men-who-have-sex-with-men and people-who-inject-drugs [2,3,4].

Moving forwards to achieving the World Health Organization (WHO) HCV elimination goals, the highly effective DAAs play a major part in obtaining elimination. Although DAAs are well tolerated and have an outstanding efficacy, in rare cases patients fail to obtain sustained virologic response (SVR). When failure occurs, this is often in the presence of resistance associated substitutions (RAS) [5,6,7,8,9].

As many patients are still in need of DAA therapy, a substantially large number of patients can fail first-generation DAA treatment [10,11]. Although newer, more robust, and pan genotypic regimens are available, failure remains present. Especially in resource limited settings where regimens are often older (more prone to resistance) and limited by the number of DAA compounds, and geno-/subtypes are more challenging [12]. RAS may jeopardize the elimination goals resulting in continuous transmission and disease progression [13].

RAS exist in two different forms: as polymorphism, or as mutations that emerge under the pharmacological pressure of the DAAs. In the former case, RAS are characteristics of that specific HCV virus, and will persist indefinitely. In the latter, reversion to wild-type could occur in a variable percentage of patients, within months in the NS3 region and years in the NS5A [14,15].

Several studies show that the existence of certain RAS can impact SVR rates and complicate retreatment options, especially among treatment experienced patients [16,17,18,19,20]. Although newer and more potent DAA regimens are available as first or second-line therapy, these are not always accessible or used [8,21]. This requires more knowledge regarding RAS to tailor first-line therapy and guide second-line therapy. Currently, available epidemiological data are scattered among different centers and therefore often generated from single center studies. Moreover, sample sizes are small. In addition, to our knowledge only a single study assessed the magnitude of RAS in a real-life cohort [22].

Without aggregated data, the identification of clinically relevant RAS circulating in the real-life population the first years after the wide availability of the DAAs, and their detailed analysis in relation to treatment data, is extremely difficult. To examine the prevalence of known and putative RAS after DAA failure, with a particular focus on RAS patterns and type of multiclass RAS, we used data from the European HepCare database from 2015 until 2019 [13]. HepCare involves multiple centers from 16 countries in Europe and the Mediterranean region.

## 2. Methods

### 2.1. Study Population

HepCare is an ongoing observational multicentre study including several clinical sites within Europe and the Mediterranean. HepCare covers 16 different countries including Belgium, Cyprus, Denmark, France, Germany, Italy, Israel, Luxembourg, the Netherlands, Poland, Portugal, Romania, Russia, Slovenia, Spain, Turkey. 

In HepCare, baseline (prior to receiving DAA treatment) or failure (after failing DAA therapy) HCV sequences were included from adults (≥18 years of age). Sequence data were combined with available clinical, virological, and demographical data and stored in a secure database. In addition, classification of genotype and subtype was performed at the clinical study site and mostly through in-house assays. As a quality control check all geno- and subtypes were reassessed using the COMET HCV subtyping tool [23].

#### Ethics Statement

All sequences were derived at the clinical study sites based on local protocols. Ethical approval for the study protocol was reviewed by the Medical Ethics Committee Erasmus MC (MEC-2018-1271).

### 2.2. Inclusion and Exclusion Criteria

For this analysis we subtracted data from HepCare of individuals, who failed DAA therapy and who had sufficient DAA treatment data available (name of treatment) from 2015 until the beginning of 2019. In addition, a viral sequence of NS3/NS5A and/or NS5B at failure should have been available covering the protein region of the failed DAA compound. We excluded failure sequences from individuals with insufficient or unknown treatment data, who discontinued treatment for several reasons (e.g., cytotoxicity, lost to follow-up, diseased) and who had a reinfection and therefore no virological failure related to resistance. 

### 2.3. Analysis of RAS

All sequences were aligned with their reference strain before analysis [24]. RAS at failure were defined as clinically relevant amino-acid changes, based on previously reported literature, at the analyzed positions compared to the reference strain [25,26,27]. In addition, to known RAS, we analyzed all amino-acid changes at clinically relevant positions across different geno- and subtypes; 36, 41, 43, 54, 55, 80, 122, 155, 156, 168, and 170 for NS3, 24, 28, 30, 31, 32, 58, 62, 92, and 93 for NS5A, and 159, 282, 316, 320, 321, 414, 448, 553, 554, 556, 558, and 559 in the NS5B region (Supplement Table S1) [25,28]. The cut-off value for variant calling was set at 15–20% for population sequencing and 5% for next generation sequencing. The overall RAS prevalence was calculated and listed with a 95% confidence interval (CI). For every gene segment (NS3, NS5a, and NS5B) our analysis was limited to patients who received an inhibitor target to that gene. In addition, we made a distinction between patients who failed an NS5B nucleoside analogue or an NS5B non-nucleoside analogue. Categorical data were statistically compared using the chi-square test or fisher’s exact, if appropriate, using R. 

## 3. Results

### 3.1. Baseline Characteristics

**Figure 1:**
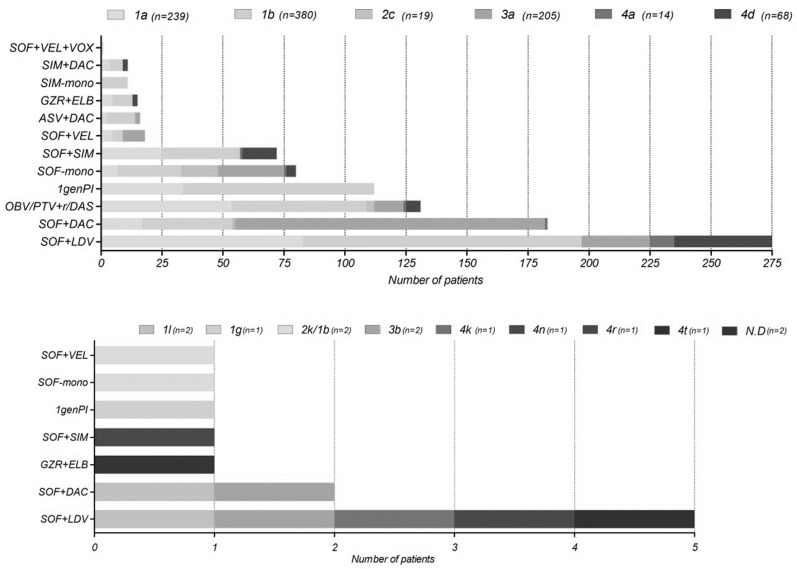
The distribution of the different direct-acting antiviral (DAA) regimens per geno—and subtype that individuals failed on. All regimens are with or without ribavirin. In the upper graph the most common genotypes are outlined. Abbreviations: N.D = non-determinant, 1genPI = first generation protease inhibitors as boceprevir and telaprevir, ASV = asunaprevir, DAC = daclatasvir, DAS = dasabuvir, ELB = elbasvir, GZR = grazoprevir, LDV = ledipasvir, OBV = ombitasvir, PTV/r = paritaprevir boosted with ritonavir, SIM = simeprevir, SOF = sofosbuvir, VEL = velpatasvir, VOX = voxilaprevir.

We included a total number of 938 individuals who failed DAA therapy. There were 59 breakthroughs, 435 relapses, 21 partial-responders, and 30 non-responders. In 393 of cases the type of virological failure was not specified by the submitter. Sequences were originated from Spain 351 (37%), Italy 225 (24%), Russia n = 168 (18%), Israel n = 102 (11%), the Netherlands n = 32 (3%), Romania n = 20 (2%), Germany n = 15 (2%), Denmark n = 14 (1%), Turkey n = 9 (1%) and France n = 2 (0.2%) (Table 1). The genotype distribution of our cohort is comparable to the European genotype distribution with a majority of genotype 1b n = 380 (40.5%), 1a n = 239 (25.4%), and 3a n = 205 (21.8%) [29]. In a lesser extent we found cases of genotype 4d n = 68 (7.2%), 4a n = 14 (1.5%), and 2c n = 19 (2%). Additionally, we identified single cases of rare subtypes as 1g, 4k, 4n, 4r, and 4t (0.1%), and two cases of subtype 1l, 2k/1b, and 3b (0.2%) (Figure 1). In two cases the subtype could not be determined (Table 2).

Most of our sequences were from male individuals n = 705 (79%). Patients had a median age of 53.7 years (interquartile range (IQR) 48.3–59.7 years) (n = 460) when their sample was taken. The median sampling time was 109 days (IQR 51–170 days) after the end of therapy (n = 283). In addition, 19 sequences were taken when patients were on DAA therapy, due to no response in viral load, and 430 patients directly after therapy (stated by the submitter as *end of* *treatment*). Clinical information regarding fibrosis stage was available for 615 individuals (66%). Most individuals had cirrhosis 367 (59%) of which 11 (2%) documented decompensated cirrhosis. Additionally, 89 (14%) were in a more advanced stage of disease METAVIR F3. Individuals who failed with lower fibrosis scores were less common in our cohort 79 (13%) with METAVIR F2, 61 (10%) with F1, and 19 (3%) with F0. 

Information regarding previous DAA therapy was available for 461 patients (49%). 397 (86%) were first time DAA failures while all others previously had received a first-generation protease inhibitor containing regimen (Figure 1). Most cases of failure were identified in patients who had received a sofosbuvir/ledipasvir (SOF + LDV) n = 280 (30%), followed by sofosbuvir/daclatasvir (SOF + DAC) n = 185 (20%) and the ombitasvir/paritaprevir/ritonavir/dasabuvir (OBV/PTV + r/DAS) regimen n = 131 (14%). Of our cohort 112 (12%) failed using the older first-generation protease inhibitors (PIs), such as boceprevir or telaprevir. DAA failure on newer regimens as grazoprevir/elbasvir (GZR + ELB) n = 16 (2%), sofosbuvir/velpatasvir (SOF + VEL) n = 19 (2%), and sofosbuvir/velpatasvir/voxilaprevir (SOF + VEL + VOX) n = 1 (0.1%) only occurred among a small number of patients (Figure 1). 

### 3.2. European Prevalence of RAS after DAA Failure 

Among the most common genotypes (n = 925) 82% of patients failed on DAA therapy with at least one RAS. Consequently, only 18% of patients failed with no RAS. Over one third of the patients (36%) had one RAS, 22% had two, and almost a quarter (24%) of the failures had three or more RAS in any of the target genes. 

**Figure 2:**
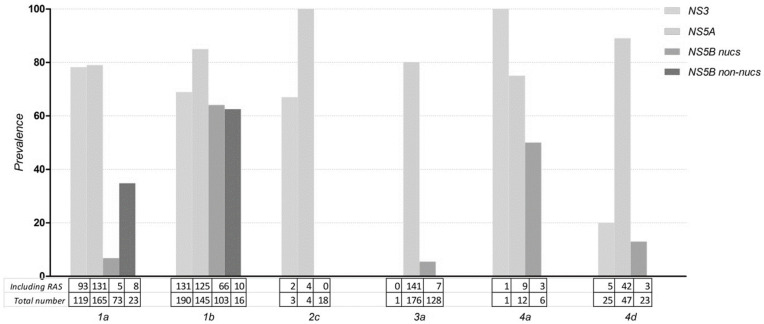
Total RAS prevalence among European failures, per different treatment region. Samples which include a RAS are divided by the total number of available sequences of that specific geno-/subtype and target region. The occurrence of RAS in rare subtypes is outlined in Table 2. No individuals with GT2c, GT3a, GT4a/d patients failed on NS5B non-nucleoside analogues with an available NS5B sequence.

NS3 RAS occurred in 0–100% of European protease-inhibitors (PI) failures and varies greatly per subtype (Figure 2). We found a lower RAS prevalence after failure in GT3a (n = 1), prevalence of 0% (95% confidence interval 0.0–79.3), and GT4d (n = 30), prevalence of 20% (8.9–39.1) compared to other genotypes (Figure 2). GT4d samples were taken with a median sampling time of 229 days (171–281 days) (n = 18). The most common RAS in GT1a were the V36M (20% (13.9–28.3) prevalence) and R155K (37% (29.1–46.3)), while for GT1b these were the Y56F (29% (23.1–35.9)) and the V170I (23% (16.9–30.6)). In GT2c failures RAS are detected at several positions not recommended by the EASL guidelines as clinically relevant: L36V (33% (6.1–79.2)), V158M (33% (6.1–79.2)), and D168V (100% (34.2–100)). Within GT3a patients no NS3 RAS were detected after failing a NS3 inhibitor (n = 1). For GT4a only one patient was treated with a PI and had an available sequence in which the Q80R RAS occurred (100% (20.7–100). For GT4d variation at position D168 was mostly common with the D168A (11% (3.1–32.8)) and the D168E (17% (5.8–39.2)) mutations (Figure 2; Supplemental Figure S1). 

In comparison to NS3 RAS the number of NS5A RAS after failing a NS5A-inhibitor was slightly higher, between 75–100%, regardless of genotype (Figure 2). In the NS5A region, GT1a NS5A-inhibitor failures predominantly harbored the Q30R mutation (34% (27.5–42) prevalence) while in GT1b the L31M (29% (22.1–37.3)) and the Y93H (73% (65.4–79.8)) were mostly present. In GT2c all patients harbored the F28C after failure (100% (51.5–100)). Similarly, as to GT1b the Y93H is the most common RAS (57% (49.7–64.2)) among GT3a failures. While in GT4a the R30L RAS (42% (19.3–68)) is most common in GT4d the T58P (57% (43.3–70.5)) and M31V (17% (8.9–30.1)) most frequently occur (Figure 2; Supplemental Figure S1).

After failing a NS5B nucleotide analogue (SOF) containing regimen, the NS5B RAS prevalence was low for all genotypes, apart from GT1b and GT4d (Figure 2). In GT1a NS5B RAS were uncommon, S282T and C316Y/R < 1.5% of failures and most commonly the L159F occurred (3% (0.5–14.9)). In GT1b we identified a substantial number of the C316N (61% (51.1–69.7)) and L159F (43.8 (33–55.2)) RAS after failure. Additionally, no S282T were detected (0% (0–3.6)). Within GT2c patients no NS5B RAS were detected after failing a SOF containing regimen (n = 18). NS5B RAS are also uncommon in GT3a L159H and S282T occurred < 1%. Additionally, the most common RAS L159F occurred with a 3% prevalence (1–8). Interestingly, the S282T was most common in GT4a (33% (12.1–64.6) and GT4d (13% (4.5–32.1) (Figure 2; Supplemental Figure S1). 

Solely for GT1a and GT1b the guidelines provide recommendations of amino-acid positions after NS5B non-nucleoside analogue failure (dasabuvir (DAS) containing regimens). In GT1a, the most common RAS were the S556G (46% (21.3–72)) and the C316Y (10% (2.8–30.1)). Similarly, as after SOF failure there was a high number of the C316N in GT1b (56% (33.2–76.9)). Additionally, the G556S RAS was common (44% (18.9–73.3)). 

### 3.3. RAS in Unusual Subtypes Defined as GT1 Non-A/B, GT3 Non-A, and GT4 Non-A/D

Although rare, several unusual subtypes were identified in our cohort. Unusual subtypes are defined as GT1 non-a/b, GT3 non-a/d, and GT4 non-a/d. In two cases the geno-subtype could not be determined (Table 2). In the first case, the submitter provided GT1a while COMET mentioned 1b and 1l. In the second case, the submitter provided GT1d while COMET identified a GT1b. 

Several RAS were detected after failure (Table 2). In addition, in unusual subtypes RAS in the NS5B region are uncommon. Sample HC_06 only had an NS3 sequence available but failed a SOF + LDV regimen. Therefore it is unclear if any RAS emerged after failure.

### 3.4. RAS Specified over DAA Regimens 

#### 3.4.1. Asunaprevir + Daclatasvir (ASV + DAC)

Although no longer used in clinical practice in Europe, in total 16 (1.7%) individuals failed (GT1a n = 3, GT1b n = 12, GT3a n = 1) an ASV + DAC regimen of which 10 relapses, two non-responders, and three breakthroughs. All GT1a patients had RAS in both NS3 + NS5A (n = 3). In one patient a V36M + Q80K + D168E and Q30E + L31C pattern emerged. In GT1b half of patients had an NS3 RAS (n = 12) and in all the patients with available NS5A sequence (n = 2) RAS were present. In NS3 the pattern of Y56F/H + D168E/V often occurred. In NS5A we identified the combination of L28G + L31M and L31M + Y93H. In the GT3 patient only the NS5A was sequenced with the A30K + P58A + A62S pattern after failure.

#### 3.4.2. First Generation Protease Inhibitors (Pis) 

Although no longer used in clinical practice, 109 (12%) of individuals failed a first generation DAAs containing either boceprevir or telaprevir. These patients were solely infected with GT1a (n = 33) and GT1b (n = 76). Among these failures there were 30 breakthroughs, 20 partial-responders, 5 non-responders, and 54 relapses. In patients who relapsed, NS3 RAS were significantly less likely to occur (65%) compared to patients with breakthroughs, partial-responders, or non-responders (88%) *p*-value = 0.01. We identified a different RAS pattern for GT1a and GT1b after failing a PI regimen with mainly the V36M (42% (27.2–59.2)) and R155K (70% (52.7–82.6)) in GT1a and the Y56F (36% (25.7–46.7)) in GT1b *p*-value < 0.001. 

#### 3.4.3. Grazoprevir + Elbasvir (GZR + ELB)

There were 16 (1.6%) individuals who failed on a GZR + ELB regimen of which there were five GT1a, eight GT1b, 2 GT4d, and one non-determinant genotype. There were seven relapses, one breakthrough, and from eight individuals the type of failure was not specified. In the breakthrough patient (GT1b) we identified a R155Q + D168N and A92V pattern. The NS5A A92V RAS did not occur in any of the other GZR + ELB failures. Most GT1a failures harboured mutations at position 30 compared to 31 + 93 in GT1b, however this was not significantly different. In GT4d no RAS were found in NS3 (n = 2), but both individuals harboured a T58P/PS RAS in the NS5A. We identified several complex NS5A RAS patterns among GZR + ELB failures as in the Q30R + H58D (n = 1) in 25% of GT1a patients which is also associated with glecaprevir + pibrentasvir (GLE + PIB) failure [30,31]. Among 13% of GT1b patients the L31M + Y93H (n = 1) and Q30R + L31M + Y93H (n = 1) were identified. Moreover, one patient harboured the Y56F + S122G (NS3) and Q30H + H58QR + Y93H (NS5A) pattern. 

#### 3.4.4. Ombitasvir + Paritaprevir + Ritonavir + Dasabuvir (OMB + PTV/r + DAS)

A total number of 131 (14%) individuals were treated with an (OMB + PTV/r + DAS) regimen of which the majority was GT1a and GT1b, 54 and 55, respectively. From the GT1a samples who were treated with a (OMB + PTV/r + DAS) regimen there were six breakthroughs, three non responders, five with a relapse, and 40 unspecified failures. Several patterns were identified in the NS3 region of GT1a failures associated with second-line protease inhibitor failure such as the Y56H + D168A (7.0% (2.4–18.6)) and Q80K + D168A (9.3% (3.7–21.6)). In the NS5A region the Q30R RAS was common after failure (48.1% (35.1–61.3)). The prevalence of the Y93H was 6.3% (2.1–16.8) and the combination of Q30H + Y93H was rare (2.1% (0.4–10.9)). In the NS5B region the A553T (9.1% (1.6–37.7)) and S556G (45.5% (21.3–72.0)) were most common. 

From the GT1b treated with an (OMB + PTV/r + DAS) regimen there were two breakthroughs, four non-responders, 27 relapses, and 22 unspecified failures. In the NS3 region the Y56H was found with a prevalence of 19.1% (10.4–32.5), the Y56F with 25.5% (15.3–39.5), and the A156V was uncommon with a prevalence of 2.1% (0.4–11.1). No P32 deletions were detected among (OMB + PTV/r + DAS) failures. RAS patterns such as the Y56H + D168A and Y56H + D168E occurred with a low prevalence of 2.2% (0.4–11.3) and the combination of Y56H + D168V slightly higher with a 13% (6.1–25.7) prevalence. In the NS5A region over half of failures had the Y93H RAS after failing a (OMB + PTV/r + DAS) regimen (65% (49.5–77.9). The combination of L31V + Y93H occurred in 8.3% (2.3–25.8). In the NS5B region all RAS identified were present at position 316 such as C316H (7.1% (1.3–31.5)), and C316N (64.3% (38.8–83.7)). 

There were three patients with a GT2c who failed a (OMB + PTV/r + DAS) regimen, from which two were non-responders. In the first sample the L36V + V158M + D168V NS3 RAS pattern was found combined with the F28C + P58S in NS5A. In the second sample the D168V RAS occurred in the NS3 region combined with the F28C + C92S in NS5A. Additionally, the third failure also had a F28C mutation in NS5A. 

GT3 failures were four non-responders, two relapses, and six underdetermined failures. RAS mostly identified were S62T (16.7% (4.7–44.8)) and Y93H (91.7% (64.6–98.5). No NS5B RAS were detected. 

Only one GT4a patient failed a (OMB + PTV/r + DAS) regimen in our cohort. No NS3 and NS5A RAS were found after failure and no NS5B sequence was available. There were six GT4d failures of which in one the Y56H + D168A and L28ALSV + M31V + T58P pattern occurred. In 83% (43.6–97.0) of failures the T58P was present. RAS at position 93 occurred with a prevalence of 33% (9.7–70) to C and CS. 

#### 3.4.5. Simeprevir + Daclatasvir (SIM + DAC) 

Although no longer used in clinical practice, this combination was used at the beginning of the NS5A-era and in our collection four patients with a GT1a infection failed on SIM + DAC. After failure, the multiclass pattern R155K (NS3) + Q30E (NS5A) pattern occurred with a prevalence of 50% (15.0–85.0) and the R155K + Q30K in 25% (4.6–69.9). In addition, in GT1b patients RAS occurred both in NS3 and NS5A after failure: Y56F + D168V + L31M + Y93H with a prevalence of 40% (11.8–76.9). Two patients with a GT4d infection failed on a SIM + DAC regimen. The first patient had a breakthrough with RAS both present in NS3 (A156G + D168E) and NS5A (L28V + R30S + T58P) after failure. The second patient relapsed with no RAS in NS3 and the M31V present in the NS5A region at failure. 

#### 3.4.6. Sofosbuvir-Mono (SOF) 

Although no longer used in clinical practice, sofosbuvir was used as a monotherapy at the beginning of the DAA-era. We analyzed 81 patients who failed a SOF + RBV regimen of which 7 GT1a, 27 GT1b, 15 GT2c, 27 GT3a, 1 GT4a, and 4 GT4d. The S282T RAS occurred only in one failure (GT3a), an overall prevalence of 1.2% (0.2–6.7). 

#### 3.4.7. Sofosbuvir + Daclatasvir (SOF + DAC)

There were 13 GT1a failures on SOF + DAC from which one non-responder, three with a relapse, and nine were undetermined failures. In NS5A most RAS were found at position Q30D/H/K/R (53.8 % (29.1–76.8)) and L31M/V (23.1% (8.2–50.3)) or a combination of both. No NS5B RAS were detected. 

GT1b were 37 failures of 30 relapses, one breakthrough, and the rest undetermined. In NS5A the L31M +Y93H and R30Q + A92K patterns were both found with a prevalence of 16.7% (3.0–56.4). In NS5B the L159F and C316N were mostly present with a prevalence of 43.3% (27.4–60.8) and 67.7% (50.1–81.4), respectively. The combination L159F + C316N occurred with 40% (24.6–57.7). 

Solely one GT2c patient failed a SOF-DAC regimen with a L31M mutation in the NS5B region and no other RAS were detected. One GT4a patient was treated with SOF + DAC, no NS5B mutations were detected and unfortunately no NS5A sequence was available. 

From the 127 GT3a failures on SOF + DAC mostly had a relapse. In NS5A the A30K occurred among 16.8% (11.3–24.3) of samples. Other RAS at position 30 were the A30S (5.6% (2.7–11.1)) and A30T (0.8% (0.1–4.4)). Only the A30S/T was combined with the Y93H. in the NS5B region RAS were only present with a low prevalence (<1%) (L159F/H, S282GR, and C316A).

One GT4a patient failed a SOF + DAC treatment. RAS were only present in the NS5A region (L30H) and none in the NS5B region. 

#### 3.4.8. Sofosbuvir + Ledipasvir (SOF + LDV) 

32% of our cohort failed on a SOF + LDV regimen. There were 83 patients with a GT1a infection. From those there was 1 non-responder, 16 with a relapse, and 66 were unspecified. Most failures harboured RAS at position 30, 31, and 58. Highly resistant RAS patterns such as, Q30R + L31M and Q30R + Y93H occurred with a prevalence of 3.7% (1.3–10.3) and 1.3% (0.2–6.7), respectively In NS5B no S282T was identified, however the L159F occurred with a prevalence of 2.6% (0.5–13.2).

There were 114 GT1b patients who failed on SOF + LDV from which, 1 non responder; 2 breakthrough, 58 relapses, and 60 unspecified failures. RAS are mostly found on position 31 and 93. The L31M had a prevalence of 32.9% (23.7–43.7), the L31I 8.5% (4.2–16.6), and the L31V 7.3% (3.4–15.1). The Y93H occurred in almost 80% of failures (79.3% (69.3–86.6) while the Y93C RAS was uncommon (1.2% (0.2–6.6). The combination of L31V + Y93H was present in 3.6% (1.2–10.0) of SOF + LDV GT1b failures. In NS5B RAS were present at the 159 and 316 positions (L159F 46.4% (29.5–64.2), C316N 61.7% (47.4–74.2).

In the GT1l sample of the patient who failed SOF + LDV only the Q30R mutation was present after failure (Table 2). 

Among GT3a failures no A30K + Y93H occurs in the NS5A region. The A30V + Y93H was rare with a prevalence of 3.6% (0.6–17.7). Other RAS were mainly present at position 62 (A/P/T/V) all with a prevalence < 10%. GT3A patients who failed an LDV-containing regimen had a significantly lower RAS prevalence than those who failed daclatasvir (DAC) (*p*-value < 0.001). In the NS5B region no RAS were detected. In the GT3b sample one RAS was detected in the NS5A region (V31M). 

Ten GT4a patients were treated with SOF/LDV. In one out of the two patients with an available NS5B sequence the S282T RAS was found. In 14% the NS5A RAS pattern L30R + L31M and L30H + Y93H occurred. 

There were 40 patients in our cohort who failed on a SOF + LDV regimen. Most failures had RAS in NS5A at position 58 (A/L/T). In 30% (10.8–60.3) The S282T or C/T was present after failure. 

#### 3.4.9. Sofosbuvir + Simeprevir (SOF + SIM)

Among GT1a patients who failed on SOF + SIM there were 11 relapses, one breakthrough, and 13 unspecified failures. Most RAS after failure were present on position 80, 155, and 170. The Q80K occurred with a prevalence of 22.7% (10.1–43.3). 

There were 32 patients with GT1b infection who failed SOM + SIM of which 15 had a relapse and the rest were unspecified. In NS3 the most identified RAS were the Y56F (22.6% (11.4–39.8)), S122T (10.3% (3.6–26.4), D168V (31.0% (17.3–49.2)), and V170I (20.7% (9.8–38.4)). In NS5B only the C316N was present with a prevalence of 37.5% (13.7–69.4). 

One patient had a breakthrough on SOF + SIM with a GT4a infection. The patient had multiclass RAS after failure as the Q80R was found in NS3 and the S282T was present in NS5B. From the GT4d patients who failed a SOF + SIM regimen (n = 14) RAS only occurred in the NS3 region and mostly at position 168 (A/E/V).

#### 3.4.10. Sofosbuvir + Velpatasvir (SOF + VEL)

There were five patients with a GT1a infection that failed SOF + VEL. From those one had a relapse. In 60% of failures the M28T mutation was present of which in 20% combined with the Q30L + Y93H and in 20% with the L31M. From only two patients the NS5B sequence was available from which in one case the C316Y RAS was present. 

There were 4 GT1b with a relapse. Unfortunately, no NS5A sequences were available. In the NS5B region 50% of samples had the L159F and in one case this was combined with the C316N. 

Among the 9 GT3a failures on SOF + VEL the A30K + Y93H occurred in 22.2% (6.3–54.7). Additionally, the A30S–Y93N NS5A pattern appeared in 11.1% (2.0–43.5). No NS5B RAS were detected. 

#### 3.4.11. Sofosbuvir + Velpatasvir + Voxilaprevir (SOF + VEL + VOX) 

Only one GT1a patient had a relapse on a SOF + VEL + VOX regimen (0.1%) with the occurrence of the Q80K in NS3 and S282T + G554C in NS5B. Unfortunately, no NS5A sequence was available. 

### 3.5. Multiclass RAS

RAS in both NS3 + NS5A occurred often in GT1a 40% (32.7–47.1) and to a similar extent in GT1b failures 48% (40.5–55.4). However, they were less common in GT2c 24% (9.6–47.3), GT3a 3.6% (0.6–17.7), GT4a 9% (1.6–37.7), and GT4d 12% (6.0–22.9).

In less than 20% of RAS in both NS3 + NS5B occurred in GT1a 16% (10.0–24.2), GT2c 0% (0–19.4), GT3a 0% (0–13.3), GT4A 13% (2.2–47.1) and GT4d 17% (6.7–35.9). Only in GT1b was there a significantly higher occurrence compared to GT1a of these multiclass RAS 32% (25.1–40.6), mostly due to the C316N (60%) in NS5B in GT1b (*p*-value = 0.03). 

The high prevalence of the C316N in GT1b also resulted in a significantly higher prevalence of NS5A + NS5B RAS in Gt1b 42% (29.0–56.7) compared to GT1a 15% (9.6–23.3) (*p*-value *=* 0.01) and GT3a 3% (1.2–7.8) (*p*-value ≤ 0.001). Additionally, there was a 33% (12.1–64.6) prevalence in GT4a and 12% (4.2–30.0) in GT4d. 

RAS in all regions occurred in 14% (8.1–21.8) of GT1a, 27% (15.7–41.9) of GT1b, and 13% (2.2–47.1) of GT4a failures. In contrast, no RAS were found in all three regions in GT2c 0% (0–18.4), GT3a 0% (0–13.3), and GT4d 0% (0–14.3). 

## 4. Discussion

Although DAAs have a high efficacy resulting in a low number of virological failures, the collaboration of several clinical and virological centres within the HepCare consortium allowed the analysis of a high number of real-life DAA failures [13]. Our results showed that RAS are highly frequent after failure and mostly occur in NS3 and NS5A regions. In various cases, complex RAS patterns were present in multiple target genes. Our results identified a great variety of RAS patterns after failure and more specific patterns over geno-/subtype. NS5A failures tend to have the highest number of RAS after failure; NS3 RAS were common, and NS5B RAS uncommon. As NS5A RAS have a very long persistence they are of the hardest potential challenge for retreatment [14,15]. However, despite frequent NS5A-RAS detection many individuals still have retreatment options. For example, individuals with the major Y93H RAS (73% GT1b and 57% GT3A), can still reach a 95% SVR rate with the SOF + VEL + VOX combination [32]. 

Most of our samples included the most common genotypes and a limited number (~1%) of unusual subtypes, defined as GT1 non a/b, GT2, GT3 non a, and GT4 non a/d. These unusual subtypes are more common in low-middle income countries, however, do appear occasionally in European clinical practices. Within our cohort there are six countries which report unusual subtypes among their patient population. Unusual subtypes are more challenging to treat as SVR rates tend to be lower potentially due to the natural variation at baseline of these subtypes [12,33,34]. Countries where these unusual subtypes are highly prevalent among the countries with the highest HCV prevalence in the world [11]. Moreover, they are low-middle income countries with limited to no resources for HCV sequencing at baseline or after failure. Therefore, aggregating available data are needed to obtain additional knowledge regarding these subtypes. 

In many cases RAS were identified which do not hamper the efficacy of second-line regimens. Moreover, significant relevant NS5A RAS patterns to these regimens (SOF + VEL + VOX or G/P) such as the Q30R + L31M and Q30R + Y93H in GT1a, the L31V + Y93H and L31V + Y93H for GT1b, and A30K + L31M and A30K/V + Y93H for GT3a all occurred with a prevalence below 5%. Nevertheless, for patients with RAS who fail a second-line regimen there are still treatment options. Recently, Dietz et al. showed that even after failure of SOF + VEL + VOX, an 81% SVR rate can be obtained using rescue regimens [35]. These results are highly promising and assuring for clinics where RAS testing after failure is not embedded in the standard of care. The newer regimens should be made available in these countries as a rescue regimen. As they are only needed for a minority of the population this should be financially feasible.

When sequencing is available and performed, interpretation of RAS after failure can be quite complicated as HCV has many geno-/subtypes and RAS patterns. Especially, as based on current knowledge and data, no algorithm is constructed to guide decision-based re-treatment. Therefore, retreatment often requires input of an experienced team. Moving forwards to elimination, this could jeopardize a simplification and decentralization of the HCV care cascade in certain regions, as the population of failures (~2–5% of 71 million who require HCV treatment) still require RAS testing and a team experienced with retreatment [11]. These regions will be the areas where the robust second-generation DAA regimens are unavailable and SOF + LDV or SOF + DAC are pan-genotypically used, or the unusual subtypes are highly epidemic. These are often also the regions where simplification and decentralization of the care cascade are highly needed to treat all the numbers of patients still infected with HCV. 

Our study has several limitations. First, due to the retrospective nature of this cohort some data is lacking, e.g., patients could have been treated elsewhere without the knowledge of the current treating physician. Moreover, individuals who failed with lower fibrosis scores were less common in our cohort (13% with F0 and F1) related to the previously installed treatment restrictions (>F2/F3 only). Secondly, most reported data are from the more difficult to treat patients, since these patients are in care of treatment centres with available HCV assays, instead of small hospitals in which DAA treatment and HCV assays were unavailable. Thirdly, failures from countries which do not have the organization or financial resources to perform sequencing are missing in our cohort. Additionally, the study cohort is based on convenience sampling and therefore the study only includes data that was shared. Consequently, not all European countries are included. Nonetheless, the genotype distribution of our cohort is comparable with the European genotype distribution and similar DAA regimens were used in Europe. Fourthly, variation of the number of RAS to other studies and in our data can be due to a different sampling time. In our study samples were drawn with a median time of 15 weeks after end of treatment. Since NS3 RAS tend to disappear mostly within a year after the end of therapy, the RAS prevalence will likely be lower when a second sample would be drawn [14]. Our study found a variable NS3 RAS prevalence among different genotypes, the lowest in GT4. Other studies varied between 53–78% [36,37]. Lastly, our study included limited data on failures of second-generation DAA therapy. However, in many countries GLE + PIB or SOF + VEL + VOX are not present or reimbursed so first-generation regimens are continuing to be used. Furthermore, our cohort reflects the available regimens in several countries. For instance, OBV/PTV + rDAS was the single available regimen in Romania for many years. Our results should, therefore, be interpreted with caution. 

Although resistance is rare, RAS prevalence after failure ranges from 0–100% depending on geno-/subtype and target region. Interpretation of the influence of RAS on retreatment is quite challenging due to the various patterns, patients’ characteristics, and previous treatment history. Moving forwards to HCV elimination, ongoing HCV resistance surveillance is essential to track the presence of RAS, RAS patterns, and gather data for a re-treatment algorithm. Additionally, more data and knowledge are required on the unusual subtypes so that the best HCV care can be provided, also in low-middle income countries. 

## Figures and Tables

**Table 1:** Cohort description of clinical available data from patients who failed direct acting antiviral therapy.

Cohort		Failure (N = 938)
**Sex** (n = 895) (%)	Male	705 (79)
**Age in years** (mean(IQR)) (n = 460)	Sample taken	53.7 (48.3–59.7)
**Type of failure,** n (%)	Breakthrough	59 (6)
	Relapses	435 (46)
	Partial-responder	21 (2)
	Non-responder	30 (3)
	Unspecified	393 (42)
**Fibrosis stage,** (n = 615) (%)	F0	19 (3)
	F1	61 (10)
	F2	79 (13)
	F3	89 (14)
	F4 unspecified	73 (12)
	F4 compensated	283 (45)
	F4 decompensated	11 (2)
**Previous therapy,** n (%)	No	179 (19)
	Yes, not with DAAs	218 (23)
	Yes, with first generations protease inhibitors	64 (7)
	Unknown	477 (51)
**Country of submission** (%)	Denmark	14 (1)
	France	2 (0.2)
	Germany	15 (2)
	Italy	225 (24)
	Israel	102 (11)
	the Netherlands	32 (3)
	Romania	20 (2)
	Russia	168 (18)
	Spain	351 (37)
	Turkey	9 (1)

**Table 2:** * Inconclusive both 1b and 1l. ** Submitted as a GT1d, however COMET classified as a 1b with a bootstrap support of 100/98/52 for the NS3/NS5A and NS5B region, respectively. *** 2k/1b according to submitter; however, COMET classified it as a GT1b subtype with a bootstrap support of 100.

Id	Subtype	Bootstrap Support	Reference Sequence	Treatment at Failure	Ns3 Ras	Ns5a Ras	Ns5b Ras
HC_01	1l	100	KC248193	SOF + DAC			No RAS
HC_02	1l	100	KC248193	SOF + LDV		R30Q	No RAS
HC_03	1g	100		1Gen PI	Q41H		
HC_04 HC_05	3b 3b	100 100	D49374 D49374	SOF + LDV SOF + DAC	Q168H	V31M	
HC_06	4k	100	EU392173	SOF + LDV			
HC_07	4n	100	FJ462441	SOF + SIM + RBV	No RAS	No RAS	No RAS
HC_08	4r	100	FJ462439	SOF + LDV		L31M, N62S	
HC_09	4t	100	FJ839869	SOF + LDV		L28M, P58H	No RAS
HC_10	N.D *	81/61/67	H77	SOF + LDV	No RAS	L31IM, H58P, E62AD	No RAS
HC_11	N.D **	100/98/52	KJ439768	GZR + ELB	No RAS	R30S, M31V, Y93S	
HC_12	1b ***			SOF + VEL		No RAS	No RAS
HC_13	1b ***			SOF + RBV			No RAS

## Data Availability

The data presented in this study are available on request from the corresponding author.

## Supplementary Materials

The following are available online at https://www.mdpi.com/article/10.3390/v14010016/s1. Table S1. Analyzed amino-acid positions for the different hepatitis C geno-/subtypes. Figure S1. distinct RAS pattern after direct-acting antiviral failure per region per Hepatitis C genotype. Table S2. European RAS prevalence after failure specified over different geno-/subtypes. Table S3. European prevalence of NS5A RAS patterns.
