# Supplementary material for: The European Prevalence of Resistance Associated Substitutions among Direct Acting Antiviral Failures
Source: Viruses. 2021 Dec 22;14(1):16. doi: 10.3390/v14010016 (PMC8781716; doi:10.3390/v14010016)
Supplement: Supplementary file 1 [file viruses-14-00016-s001.zip › viruses-1486074-supplementary.pdf]

Table S1: Analysed amino-acid positions for the different hepatitis C geno- and subtype.

Amino-acid positions		Geno-and subtype					
drug class (genome region) / position		1a	1b	2c	3a	4a	4d
**Protease inhibitor (NS3)**	*36*	V36[24]	V36[24]	*L36¥*			
	41		Q41[24]				
	43	F43[26]	F43[24]				
	54	T54[24]	T54[24]				
	55		V55[24]				
	56	Y56[24]	Y56[24]	F56[24]	Y56[24]	Y56[24]	Y56[24]
	80	Q80[24]	Q80[24]		Q80[26,28]	Q80[24]	Q80[24]
	122	S122[24]	S122[24]				
	155	R155[24]	R155[24]			R155[24]	R155[24]
	156	A156[24]	A156[24]	A156‡[29]	A156[24]	A156[24]	A156[24]
	158	V158[24]	V158[24]				
	168	D168[24]	D168[24]	D168‡[29]	Q168[24]	D168[24]	D168[24]
	170	I170[24]	V170[24]				
**NS5A inhibitors (NS5A)**	*24*	K24[24]					
	28	M28[24]	L28[24]	F28[24]	M28[24]	L28[24]	L28[24]
	30	Q30[24]	R30[24]	K30[24]	A30[24]	L30[24]	R30[24]
	31	L31[24]	L31[24]	L31[24]	L31[24]	M31[24]	M31[24]
	32	P32[24]					
	38	S38[24]					
	58	H58[24]	P58[24]	P58[28]	P58[30,31]	P58[24]	T58[24]
	62		Q/E62D		S62L	E62	E62
	92	A92[24]	A92[24]	C92[24]	E92[24]		
	93	Y93[24]	Y93[24]	Y93[24]	Y93[24]	Y93[24]	Y93[24]
**Nucleotide analogue (NS5B)**	*159*	L159[24]	L159[24]	L159[24]	L159[24]		
	282	S282[24]	S282[24]	S282[24]	S282[24]	S282[24]	S282[24]
	316	C316F/R/Y[24]	C316N/F/H[21]			*C316Y	
	320	L320[24]					
	321	V321[24]			V321[24]		
**Non-Nucleoside analogue (NS5B)**	*316*	C316Y[21]	C316H/N/Y/W[21]				
	414	M414[24]	M414[24]				
	444	D/N444					
	448	Y448[24]	Y448[24]				
	553	A553[24]	A553[24]				
	554	G554[24]	G554[24]				
	556	S556[24]	S556[24]				

	558	G558[24]	G558[24]	
	559	D559[24]	D559[24]	

Table 1. Covered amino-acid positions for treatment- emerging RAS per geno-and subtype
¥ Possible treatment emerging mutation found
‡ Positions indicated as clinically relevant in genotype 2a and 2b; unknown for genotype 2c

Figure S1. Figure distinct RAS pattern after direct-acting antiviral failure per region per Hepatitis C genotype.

In NS3 there was no data or patients who failed with a GT3a and GT4a HCV infection. In NS5B there was no data of patients who failed with a GT2c HCV infection. In the NS5B region there is a distinction between patients who failed a SOF or DAS containing regimen.

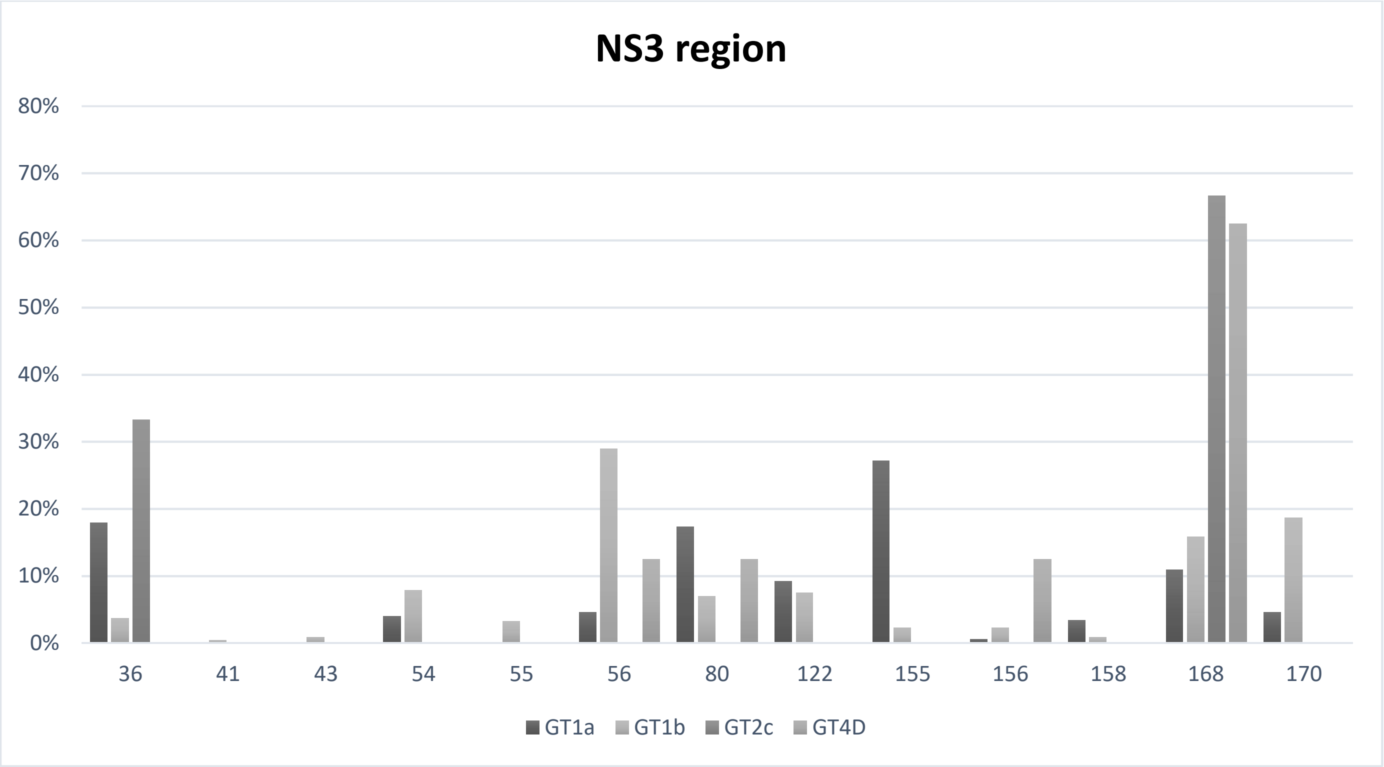

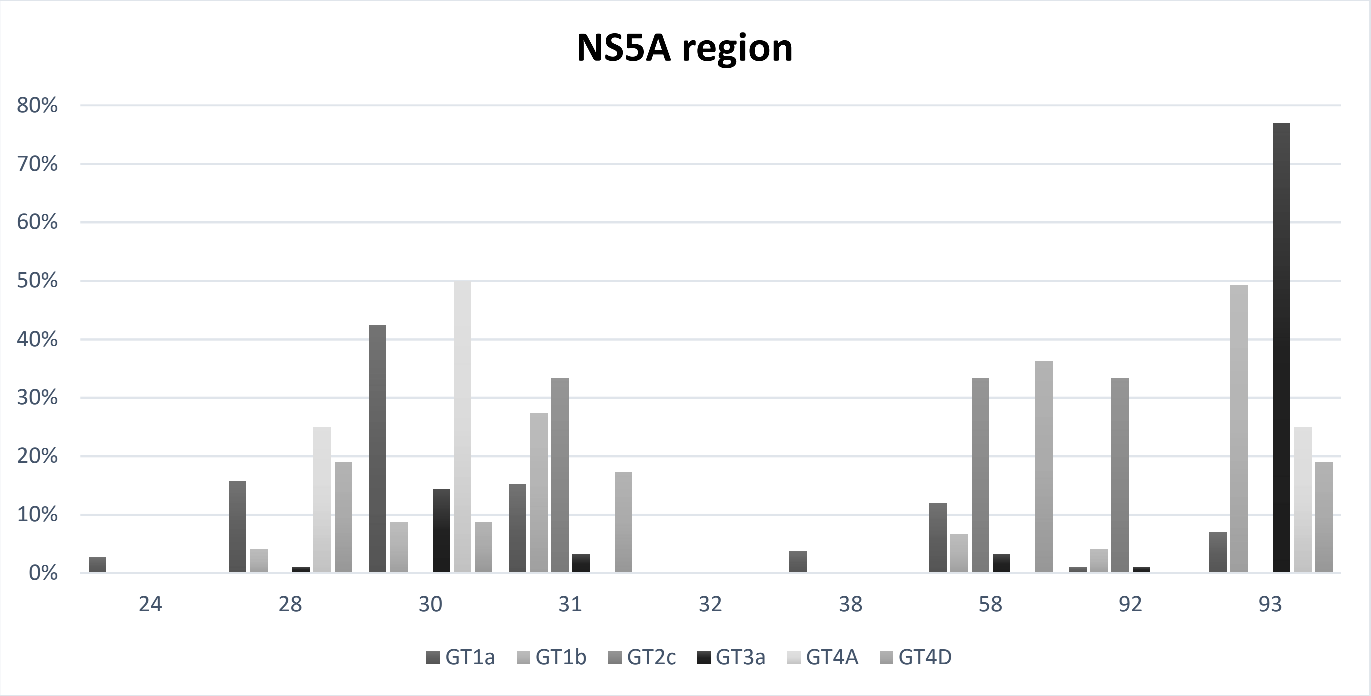
NS5A region
80%
70%
60%
50%
40%
30%
20%
10%
0%
24
28
30
31
32
38
58
92
93
GT1a
GT1b
GT2c
GT3a
GT4A
GT4D

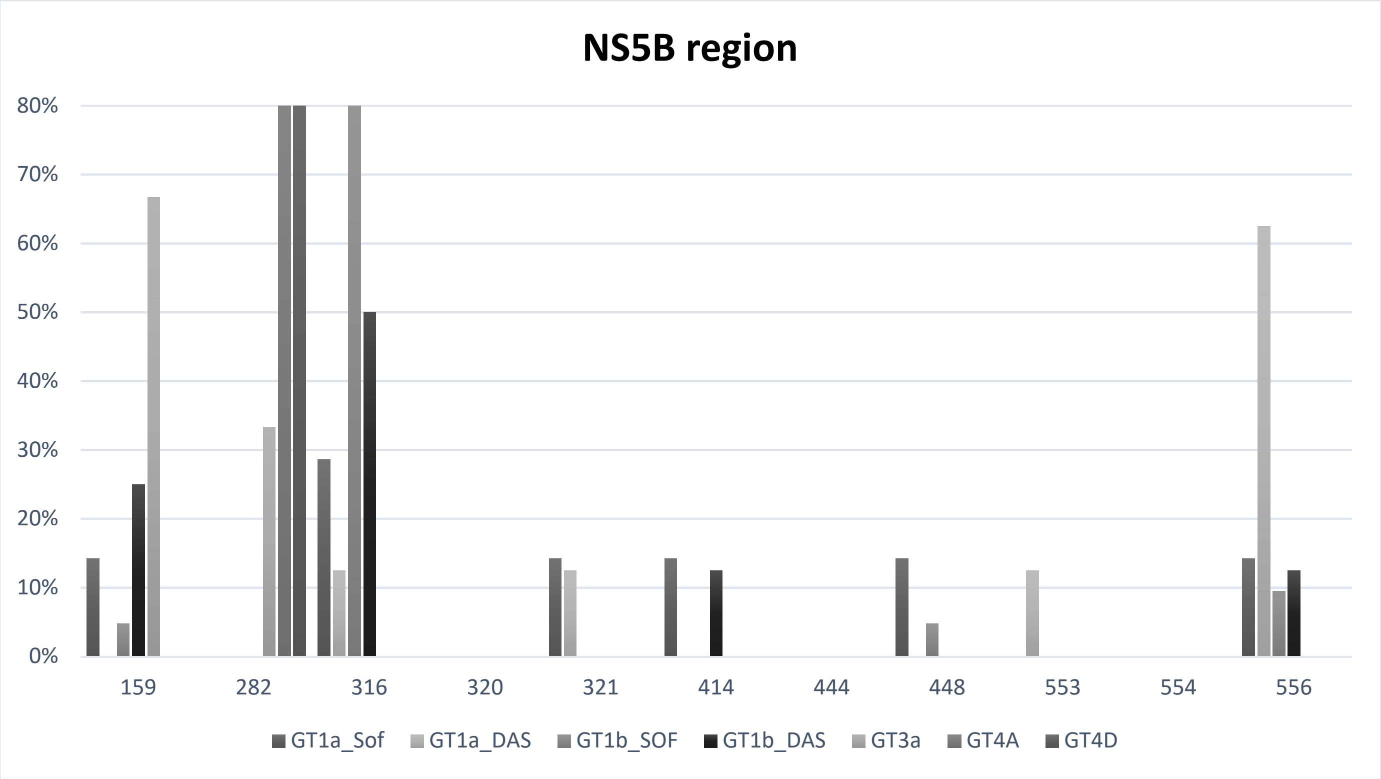
NS5B region
80%
70%
60%
50%
40%
30%
20%
10%
0%
159
282
316
320
321
414
444
448
553
554
556
GT1a_Sof
GT1a_DAS
GT1b_SOF
GT1b_DAS
GT3a
GT4A
GT4D

Table S2. European RAS prevalence after failure specified over different geno-/subtypes.

HCV genotype 1a			
**NS3**	**Prevalence**	*95% Confidence interval*	
V36L	5.9	2.9	11.6
V36M	20.2	13.9	28.3
T54A	1.7	0.5	5.9
T54S	4.2	1.8	9.5
Y56H	5.9	2.9	11.6
Y56W	0.8	0.1	4.6
Q80H	0.8	0.1	4.6
Q80K	20.2	13.9	28.3
Q80KN	0.8	0.1	4.6
Q80L	5.0	2.3	10.6
S122G	11.0	6.6	17.9
S122I	0.8	0.1	4.6
S122N	1.7	0.5	6.0
R155I	0.8	0.1	4.6
R155K	37.3	29.1	46.3
R155KT	0.8	0.1	4.6
R155T	1.7	0.5	6.0
A156V	0.9	0.2	4.7
V158G	0.9	0.2	4.7
V158I	1.7	0.5	6.1
V158M	1.7	0.5	6.1
V158L	0.9	0.2	4.7
D168A	5.7	2.6	11.8
D168AV	0.9	0.2	5.2
D168E	3.8	1.5	9.3
D168V	4.7	2.0	10.6
D168Y	1.9	0.5	6.6
I170T	1.9	0.5	6.7
I170V	5.7	2.6	11.9
**NS5A**	**Prevalence**	*95% Confidence interval*	
K24E	0.6	0.1	3.6
K24Q	0.6	0.1	3.6
K24R	1.3	0.4	4.6
K24S	0.6	0.1	3.6
M28L	0.6	0.1	3.5
M28T	10.0	6.2	15.6
M28PT	0.6	0.1	3.5
M28V	7.5	4.3	12.7
Q30A	0.6	0.1	3.5
Q30D	0.6	0.1	3.5
Q30E	3.1	1.3	7.1

Q30H	6.9	3.9	11.9
Q30HR	0.6	0.1	3.5
Q30K	1.9	0.6	5.4
Q30L	1.3	0.3	4.4
Q30R	34.4	27.5	42.0
L31C	0.6	0.1	3.4
L31F	0.6	0.1	3.4
L31M	14.9	10.2	21.2
L31V	1.9	0.6	5.3
S38APT	0.6	0.1	3.4
S38F	1.2	0.3	4.3
S38H	0.6	0.1	3.4
S38P	0.6	0.1	3.4
S38Y	0.6	0.1	3.4
S38W	0.6	0.1	3.4
H58C	0.6	0.1	3.4
H58D	3.1	1.3	7.0
H58G	0.6	0.1	3.4
H58QR	0.6	0.1	3.4
H58N	0.6	0.1	3.4
H58P	6.8	3.8	11.7
H58Y	1.2	0.3	4.4
A92P	0.6	0.1	3.5
A92T	0.6	0.1	3.5
Y93C	1.3	0.4	4.6
Y93H	7.1	4.0	12.2
Y93N	0.6	0.1	3.5
**NS5B**	**Prevalence**	***95% Confidence interval***	
L159F	2.9	0.5	14.9
S282T	1.4	0.3	7.7
*C316N**	1.4	0.2	7.6
*C316R**	1.4	0.2	7.6
*C316Y**	1.4	0.2	7.6
*C316Y***	10.0	2.8	30.1
V321F	1.4	0.2	7.6
A553T	9.1	1.6	37.7
S556G	45.5	21.3	72.0

*Sofosbuvir; **Dasabuvir

HCV genotype 1b

NS3	*Prevalence*	95% Confidence interval	
V36A	1.1	0.3	3.8
V36C	0.5	0.1	3.0
V36L	0.5	0.1	3.0
V36M	1.1	0.3	3.8
F43S	0.5	0.1	3.0
T54A	4.8	2.5	8.8

T54S	4.8	2.5	8.8
V55A	1.1	0.3	3.8
V55F	0.5	0.1	2.9
Y56F	29.1	23.1	35.9
Y56H	6.3	3.7	10.8
Q80K	0.5	0.1	2.9
Q80L	2.1	0.8	5.3
Q80R	3.2	1.5	6.8
S122N	1.7	0.6	4.8
S122R	0.6	0.1	3.1
S122T	7.3	4.3	12.1
R155A	0.6	0.1	3.1
R155G	0.6	0.1	3.1
R155Q	1.7	0.6	4.8
R155W	0.6	0.1	3.1
A156G	0.6	0.1	3.1
A156S	1.1	0.3	4.0
A156T	1.7	0.6	4.8
A156V	0.6	0.1	3.1
V158I	0.6	0.1	3.1
D168A	3.4	1.6	7.3
D168E	6.3	3.6	11.0
D168H	0.6	0.1	3.2
D168N	0.6	0.1	3.2
D168V	14.4	9.9	20.3
V170A	2.8	1.1	7.0
V170I	23.1	16.9	30.6
V170T	0.7	0.1	3.9
**NS5A**	**Prevalence**	**95% Confidence interval**	
L28G	0.8	0.1	4.2
L28M	4.6	2.1	9.6
L28V	0.8	0.1	4.2
R30AGP	0.8	0.1	4.1
R30K	0.8	0.1	4.1
R30Q	12.0	7.5	18.6
L31EV	0.7	0.1	4.1
L31F	0.7	0.1	4.1
L31I	6.0	3.1	11.3
L31IM	0.7	0.1	4.1
L31M	29.1	22.1	37.3
L31V	6.0	3.1	11.3
P58A	1.4	0.4	5.0
P58S	4.9	2.4	9.8
P58T	2.1	0.7	6.0
A92E	0.7	0.1	3.9
A92K	0.7	0.1	3.9
A92T	0.7	0.1	3.9

A92V	3.5	1.5	8.0
Y93C	0.7	0.1	3.9
Y93H	73.2	65.4	79.8
**NS5B**	**Prevalence**	**95% Confidence interval**	
L159F	43.8	33.0	55.2
S282	0.0	0.0	3.6
*C316N**	60.8	51.1	69.7
*C316N***	56.3	33.2	76.9
C316H	6.3	1.1	28.3
M414I	9.1	1.6	37.7
G556S	44.4	18.9	73.3

*Sofosbuvir; **Dasabuvir

HCV genotype 2c

**NS3**	**Prevalence**	**95% Confidence interval**	
L36V	33.3	6.1	79.2
V158M	33.3	6.1	79.2
D168V	100	34.2	100.0
**NS5A**	**Prevalence**	**95% Confidence interval**	
F28C	100	51.0	100.0
L31M	25	4.6	69.9
P58S	25	4.6	69.9
C92S	25	4.6	69.9

There were no NS5B failures or NS5B sequences available in our cohort of GT2c failures

HCV genotype 3a

**NS5A**	**Prevalence**	**95% Confidence interval**	
M28L	0.6	0.1	3.2
A30K	14.9	10.3	20.9
A30S	5.1	2.7	9.5
A30T	0.6	0.1	3.2
A30V	1.1	0.3	4.1
L31F	0.6	0.1	3.1
L31I	1.7	0.6	4.9
L31M	0.6	0.1	3.1
P58A	1.1	0.3	4.1
P58R	0.6	0.1	3.2
P58S	2.3	0.9	5.7
P58T	1.1	0.3	4.1
E92A	0.6	0.1	3.2
E92del	0.6	0.1	3.2
Y93H	57.1	49.7	64.2
Y93N	0.6	0.1	3.2
**NS5B**	**Prevalence**	**95% Confidence interval**	
L159F	2.8	1.0	8.0

L159H	0.9	0.2	5.2
E237G	0.9	0.2	4.9
S282GR	0.8	0.1	4.4
S282T	0.8	0.1	4.4
*C316A**	0.8	0.1	4.3
V321A	0.8	0.1	4.3

*Sofosbuvir. There were no NS3 failures or NS3 sequences available in our cohort of GT3a failures. No RAS were detected at position 321 of GT3a DAS failures

HCV genotype 4a

**NS3**	**Prevalence**	**95% Confidence interval**	
Q80R	1	20.7	100
**NS5A**	**Prevalence**	**95% Confidence interval**	
L28M	25.0	8.9	53.2
L28V	33.3	13.8	60.9
R30F	8.3	1.5	35.4
R30H	25.0	8.9	53.2
R30L	41.7	19.3	68.0
P58T	8.3	1.5	35.4
E62Q	9.1	1.6	37.7
Y93H	8.3	1.5	35.4
**NS5B**	**Prevalence**	**95% Confidence interval**	
S282T	33.33	12.1	64.6

HCV genotype 4d

**NS3**	**Prevalence**	**95% Confidence interval**	
Y56H	4.2	0.7	20.2
A156G	5.6	1.0	25.8
D168A	11.1	3.1	32.8
D168E	16.7	5.8	39.2
D168V	5.6	1.0	25.8
**NS5A**	**Prevalence**	**95% Confidence interval**	
L28ALSV	2.1	0.4	11.1
L28M	6.4	2.2	17.2
L28S	2.1	0.4	11.1
L28V	12.8	6.0	25.2
R30C	2.1	0.4	11.1
R30H	2.1	0.4	11.1
R30S	2.1	0.4	11.1
M31I	2.1	0.4	11.1
M31V	17.0	8.9	30.1
T58A	6.4	2.2	17.2
T58L	2.1	0.4	11.1
T58LP	2.1	0.4	11.1
T58P	57.4	43.3	70.5

T58PS	2.1	0.4	11.1
E62M	2.2	0.4	11.6
E62Q	2.2	0.4	11.6
Y93C	8.7	3.4	20.3
Y93CS	2.2	0.4	11.3
Y93H	8.7	3.4	20.3
**NS5B**	**Prevalence**	**95% Confidence interval**	
S282CT	8.7	2.4	26.8
S282T	4.3	0.8	21.0

Table S3. European prevalence of NS5A RAS patterns.

**HCV genotype 1a**	***Prevalence***	***95% Confidence interval***	
H58P+A92AP	*0,6*	*0,1*	*3,6*
K24Q+M28L+H58P+Y93H	*0,6*	*0,1*	*3,6*
K24R+Q30H	*0,6*	*0,1*	*3,6*
K24R+Q30R	*0,6*	*0,1*	*3,6*
K24S+Q30R+Y93H	*0,6*	*0,1*	*3,6*
L31F+H58N	*0,6*	*0,1*	*3,6*
L31M+H58P	*1,9*	*0,7*	*5,5*
L31V+H58P	*0,6*	*0,1*	*3,6*
M28MT+Q30QR	*0,6*	*0,1*	*3,6*
M28MT+Q30QR+H58Y	*0,6*	*0,1*	*3,6*
M28MT+Q30R+H58P	*0,6*	*0,1*	*3,6*
M28MV+Q30R	*0,6*	*0,1*	*3,6*
M28MV+S38APST	*0,6*	*0,1*	*3,6*
M28T+H58P+S38F	*0,6*	*0,1*	*3,6*
M28T+Q30R	*1,9*	*0,7*	*5,5*
M28V+L31M	*0,6*	*0,1*	*3,6*
M28V+Q30R	*1,9*	*0,7*	*5,5*
M28V+Q30R+S38SY	*0,6*	*0,1*	*3,6*
Q30E+L31C	*0,6*	*0,1*	*3,6*
Q30E+S38W	*0,6*	*0,1*	*3,6*
Q30H+H58C+Y93H	*0,6*	*0,1*	*3,6*
Q30H+H58HQR+Y93H	*0,6*	*0,1*	*3,6*
Q30H+H58Y	*0,6*	*0,1*	*3,6*
Q30H+Y93H	*0,6*	*0,1*	*3,6*
Q30KQ+A92AT+Y93HY	*0,6*	*0,1*	*3,6*
Q30R+H58D	*1,9*	*0,7*	*5,5*
Q30R+L31LM+H58DH	*0,6*	*0,1*	*3,6*
Q30R+L31M	*2,6*	*1,0*	*6,4*
Q30R+L31V	*0,6*	*0,1*	*3,6*
Q30R+S38FS	*0,6*	*0,1*	*3,6*
Q30R+S38P	*0,6*	*0,1*	*3,6*

HCV genotype 1b	*Prevalence*	*95% Confidence interval*	
L31M+Y93H	*16,0*	*10,1*	*23,9*
L28M+Y93H	*3,1*	*1,0*	*7,8*
P58PS+Y93H	*3,1*	*1,0*	*7,8*
R30Q+Y93H	*3,1*	*1,0*	*7,8*
A92V+Y93H	*2,3*	*0,6*	*6,7*
L31I+Y93H	*2,3*	*0,6*	*6,7*
L31V+Y93H	*3,1*	*1,0*	*7,8*
R30Q+L31M+Y93H	*2,3*	*0,6*	*6,7*
R30Q+L31I+Y93H	*1,5*	*0,3*	*5,5*
L28G+L31I	*0,8*	*0,0*	*4,3*
L28M+R30AGP+L31F+A92E+Y93H	*0,8*	*0,0*	*4,3*
L28M+L31M+Y93H	*0,8*	*0,0*	*4,3*
L28V+Y93H	*0,8*	*0,0*	*4,3*
L31EV+Y93H	*0,8*	*0,0*	*4,3*
L31I+A92T+Y93H	*0,8*	*0,0*	*4,3*
L31IM+Y93H	*0,8*	*0,0*	*4,3*
L31LV+Y93HY	*0,8*	*0,0*	*4,3*
L31M+A92V+Y93H	*0,8*	*0,0*	*4,3*
L31M+P58S+Y93C	*0,8*	*0,0*	*4,3*
L31V+P58T+Y93H	*0,8*	*0,0*	*4,3*
P58A+Y93H	*0,8*	*0,0*	*4,3*
P58T+Y93H	*0,8*	*0,0*	*4,3*
R30K+P58A+Y93H	*0,8*	*0,0*	*4,3*
R30Q+L31M	*0,8*	*0,0*	*4,3*
R30Q+L31M+P58S+Y93H	*0,8*	*0,0*	*4,3*
R30Q+L31V+Y93H	*0,8*	*0,0*	*4,3*
R30Q+P58S+Y93H	*0,8*	*0,0*	*4,3*
R30Q+A92K	*0,8*	*0,0*	*4,3*
Q30R+L31M	*3,1*	*1,0*	*7,8*
L31M+H58P	*2,3*	*0,6*	*6,7*
M28T+Q30R	*2,3*	*0,6*	*6,7*
M28V+Q30R	*2,3*	*0,6*	*6,7*
Q30R+H58D	*2,3*	*0,6*	*6,7*
H58P+A92AP	*0,8*	*0,0*	*4,3*
K24Q+M28L+H58P+Y93H	*0,8*	*0,0*	*4,3*
K24R+Q30H	*0,8*	*0,0*	*4,3*
K24R+Q30R	*0,8*	*0,0*	*4,3*
K24S+Q30R+Y93H	*0,8*	*0,0*	*4,3*
L31F+H58N	*0,8*	*0,0*	*4,3*
L31V+H58P	*0,8*	*0,0*	*4,3*
M28MT+Q30QR	*0,8*	*0,0*	*4,3*
M28MT+Q30QR+H58Y	*0,8*	*0,0*	*4,3*
M28MT+Q30R+H58P	*0,8*	*0,0*	*4,3*
M28MV+Q30R	*0,8*	*0,0*	*4,3*
M28MV+S38APST	*0,8*	*0,0*	*4,3*

M28T+H58P+S38F	*0,8*	*0,0*	*4,3*
M28V+L31M	*0,8*	*0,0*	*4,3*
M28V+Q30R+S38SY	*0,8*	*0,0*	*4,3*
Q30E+L31C	*0,8*	*0,0*	*4,3*
Q30E+S38W	*0,8*	*0,0*	*4,3*
Q30H+H58C+Y93H	*0,8*	*0,0*	*4,3*
Q30H+H58HQR+Y93H	*0,8*	*0,0*	*4,3*
Q30H+H58Y	*0,8*	*0,0*	*4,3*
Q30H+Y93H	*0,8*	*0,0*	*4,3*
Q30KQ+A92AT+Y93HY	*0,8*	*0,0*	*4,3*
Q30R+L31LM+H58DH	*0,8*	*0,0*	*4,3*
Q30R+L31V	*0,8*	*0,0*	*4,3*
Q30R+S38FS	*0,8*	*0,0*	*4,3*
Q30R+S38P	*0,8*	*0,0*	*4,3*
**HCV genotype 2c**	***Prevalence***	***95% Confidence interval***	
F28C+P58S	*25,0*	*4,6*	*69,9*
F28C+C92S	*25,0*	*4,6*	*69,9*
F28C+L31M	*25,0*	*4,6*	*69,9*
**HCV genotype 3a**	***Prevalence***	***95% Confidence interval***	
A30T+Y93H	*0,9*	*0,2*	*5,2*
M28L+A30S+Y93H	*0,9*	*0,2*	*5,2*
P58S+Y93H	*0,9*	*0,2*	*5,2*
L31I+Y93H	*0,9*	*0,2*	*5,2*
A30S+Y93H	*0,9*	*0,2*	*5,2*
A30K+E92A	*0,9*	*0,2*	*5,2*
A30V+Y93H	*0,9*	*0,2*	*5,2*
A30K+Y93H	*1,9*	*0,5*	*6,6*
P58R+Y93H	*0,9*	*0,2*	*5,2*
**HCV genotype 4a**	***Prevalence***	***95% Confidence interval***	
L28M+L30R	*25,0*	*4,6*	*69,9*
**HCV genotype 4d**	***Prevalence***	***95% Confidence interval***	
L28LV+R30H	*2,2*	*1,9*	*9,8*
M31V+T58P	*6,5*	*5,9*	*13,9*
M31V+T58A	*2,2*	*1,9*	*9,8*
T58P+Y93CS	*2,2*	*1,9*	*9,8*
T58P+Y93C	*2,2*	*1,9*	*9,8*
R30C+Y93H	*2,2*	*1,9*	*9,8*
T58P+Y93H	*4,3*	*3,8*	*11,9*
L28M+T58P	*6,5*	*5,9*	*13,9*
R30H+T58A	*2,2*	*1,9*	*9,8*
T58P+Y93CY	*2,2*	*1,9*	*9,8*
T58A+Y93C	*2,2*	*1,9*	*9,8*
M31V+Y93H	*2,2*	*1,9*	*9,8*
L28M+T58H	*2,2*	*1,9*	*9,8*
L28V+R30S+T58P	*2,2*	*1,9*	*9,8*
L28ALSV+M31MV+T58P	*2,2*	*1,9*	*9,8*

L28S+T58P+Y93C	*2,2*	*1,9*	*9,8*
L28V+M31IM+T58L	*2,2*	*1,9*	*9,8*
